# Relationship between Serum Levels of Anti-Mullerian Hormone, Adiponectin and Oxidative Stress Markers in Patients with Polycystic Ovary Syndrome

**DOI:** 10.22074/ijfs.2020.5809

**Published:** 2020-02-25

**Authors:** Mozhgan Kohzadi, Mohammad Rasool Khazaei, Farzaneh Choobsaz, Mozafar Khazaei

**Affiliations:** 1 Students Research Committee, Kermanshah University of Medical Sciences, Kermanshah, Iran; 2 Fertility and Infertility Research Center, Health Technology Institute, Kermanshah University of Medical Sciences, Kermanshah, Iran

**Keywords:** Adiponectin, Anti-Mullerian Hormone, Polycystic Ovary Syndrome

## Abstract

**Background:**

Polycystic ovary syndrome (PCOS) is the most common endocrine disorder in women of reproductive age. Anti-Mullerian hormone (AMH) is a valid indicator of ovarian function and is used for PCOS diagnosis. Some studies have shown that adipokines affect the synthesis of AMH, and therefore they are somehow related in function. The aim of the present study was to determine the relationship between serum levels of AMH, adiponectin and oxidative stress markers in PCOS patients.

**Materials and Methods:**

In this cross-sectional study, PCOS patients and healthy women (80 cases in total) were investigated. Serum levels of AMH, adiponectin, gonadotropins, androgens, total antioxidant capacity (TAC), nitric oxide (NO) and insulin resistance (IR) were measured by standard methods. An independent t test was used to compare the two groups and Pearson correlation coefficient was used to determine the relationship between variables.

**Results:**

There was a significant difference between the means of AMH (5.16 ± 5.3 vs. 2.44 ± 2.5 ng/mL) (P=0.007) and adiponectin (24.55 ± 9.41 vs. 30.57 ± 14.2 µg/L) (P=0.029) among the PCOS and control groups, respectively. The correlation between AMH and adiponectin in the control group was statistically significant and negative (P=0.028, r=-0.35), while in the PCOS group it was not significant (P=0.11, r=-0.25).

**Conclusion:**

Various biochemical and hormonal factors differ between PCOS and healthy women. Different factors can influence AMH and adiponectin levels independently of PCOS in women of reproductive age.

## Introduction

Polycystic ovary syndrome (PCOS) is a metabolic disorder and one of the most common endocrine disorders in women of reproductive age, with an incidence of 4-18% (1). This syndrome is the main cause of anovulation in infertile women. Although PCOS was initially recognized by increasing androgen secretion from adrenal glands and ovaries, hirsutism, irregular menstruation, large ovaries, increased number of primary and pre-anteral ovarian follicles, and disturbances in the dominant follicle selection, today it is introduced as a disorder with multiple causes and metabolic consequences (2). However, the pathogenesis of PCOS is complex and not completely understood. Previous studies have shown that androgens and insulin play key roles in the development of this disease (3, 4). PCOS patients have higher serum levels of testosterone and insulin, triglycerides, cholesterol, and lower serum levels of sex hormone-binding globulin (SHBG) and follicle stimulating hormones (FSH) compared to healthy women (4, 5). Many studies have found effective oxidative stress in the pathogenesis of anovulation, insulin resistance (IR), and hyperandrogenism in PCOS patients (6). Also, signs of high serum levels of oxidative stress, such as malondialdehyde (MDA) and reduction of total antioxidant capacity (TAC) have been observed in PCOS patients (7).

Anti-Mullerian hormone (AMH) is a glycoprotein from the family of transforming growth factor-beta (TGF-β), secreted by granulosa cells of the antral follicles (4-6 mm). AMH secretion gradually decreases during follicular growth and cannot be distinguished in follicles larger than 8 mm. Currently the serum level of AMH, as a valid indicator of ovarian function, is determined in women’s fertility screening and PCOS diagnosis, allowing for targeted treatment of infertility (8). The concentration of AMH is related to the number of small follicles and ovarian reserve (6, 9). The number of the small follicles is relatively constant during the menstrual cycle and it seems that AMH concentration has insignificant fluctuation during this time. As age increases, AMH decreases gradually, indicating a decrease in the number of ovarian follicles and reaching the menopausal stage (9).

AMH has an inhibitory effect on the growth of primordial follicles, thus preventing them from finishing early in the life of a woman (10, 11). In PCOS women, the number of small follicles (2-5 mm) is 2 to 3 times that of healthy women, which leads to an increase in the concentration of AMH in these individuals (12, 9) and it seems that AMH concentration is effective in the pathogenesis of PCOS and anovulation. The increased AMH reduces the sensitivity of the antral follicles to the follicle-stimulating hormone (FSH) and subsequently prevents both the selection of the dominant follicle and the growth of follicles in the antral phase (13). Also AMH inhibits the aromatase enzyme, leading to a decrease in the production of follicular estradiol, which in turn may be accompanied by a defect in the selection of the dominant follicle (14).

Nutritional status and obesity may affect the synthesis of AMH, as some studies have reported a decrease in AMH levels in obese women, indicating a negative correlation between AMH and BMI, while others have not mentioned a correlation between nutritional factors, body mass index (BMI) and AMH (14, 15). The prevalence of obesity is more than 50% in patients with PCOS, leading to IR and increased insulin levels in these patients. Obesity may contribute to the clinical complications of PCOS, and hyperinsulinemia can be associated with the termination of ovarian follicle growth (16).

In addition to energy storage, adipose tissue can synthesize and secrete important metabolic proteins, including adipokines that regulate multiple biological actions (17). An adipokine, which accounts for about 0.01% of plasma proteins, is adiponectin (18). This protein has two receptors (ADIPO R1 and ADIPO R2) and pivotal roles in lipid metabolism, such as increasing insulin sensitivity and employing anti-inflammatory effects (19). Several studies have shown that there is correlation between adiponectin deficiencies in adipose tissue and the reduction of ovarian reserve in obese PCOS and non-PCOS women (9, 20). Some studies have reported adiponectin reduction in PCOS patients, which may be due to obesity and IR (21). Also, it has been suggested that leptin and not adiponectin may affect the synthesis of AMH in women. It seems that there is a negative correlation between insulin and AMH levels, while there is a positive correlation between AMH and adiponectin (22).

Undoubtedly, the recognition of the factors involved in the pathogenesis of PCOS and how they interferes with the syndrome can lead to a better understanding of PCOS and therefore provides access to appropriate methods for its diagnosis and treatment. Regarding the importance of AMH, the prevalence of obesity and related dysfunction of adiponectin in PCOS, the aim of present study was to determine the correlation between AMH, adiponectin and oxidative stress markers in PCOS patients.

## Materials and Methods

### Study subjects

In this cross-sectional study, 40 PCOS patients and 40 healthy women aged 18-40 years were randomly divided and evaluated in two groups. The sample size was accepted by an academic static consult in related committee. PCOS and healthy subjects were selected by our gynecologist from her private clinic. The diagnosis of PCOS was done based on Rotterdam Criteria (23). Exclusion criteria were: subjects with diabetes, or underlying systemic disease, galactorrhea, any endocrine disease associated with thyroid stimulating hormone (TSH), prolactin or 17α-hydroxy progesterone levels, usage of drugs that affect the function of the hypothalamus-pituitary-ovarian axis or insulin-sensitizing drugs such as metformin during last three months and using contraceptives during last 4 weeks. Also, women with addiction to cigarette, narcotics or alcohol, as well as women who were involved in regular exercise activities during the study period were excluded. This study was approved by the Ethics Committee of Kermanshah University of Medical Sciences (KUMS.REC.1395.626) and the patients signed informed consent.

### Sample collection

Blood samples were collected in similar conditions for each participant on the 3rd and 5th days of their menstrual cycle and after 8 hours of fasting. AMH Enzyme-linked immunosorbent assay (ELISA, Beckman Coulter, USA) was performed according to manufacturer’s instructions. Adiponectin, gonadotropins and androgen were detected by chemiluminescence technique (Immulite 2000, Siemens, Germany). To evaluate IR, the HOMA-IR index (Homeostasis Model Assessment for IR) was used as follows: fasting blood glucose (mmol/L) concentration x fasting insulin (μIU/mL) divided by constant 22.5; an index > 2 indicated IR (21).

### Ferric reducing antioxidant power assay

The TAC of the sera was assessed by Ferric reducing antioxidant power assay (FRAP) method. Briefly, serum (150 μl) was mixed with 1.5 ml of fresh FRAP reagent (10 mM 2, 4, 6- Tripyridyl-s-Triazine, 20 mM Fecl_3_, 6H_2_O solution and 300 mM acetate buffer pH=3.6), and incubated at 37˚C for 10 minutes. Then the absorbance was measured at 593 nm using a spectrophotometer (Pharmacia, Novaspec II, Biochrom, England) and was compared to a standard curve constructed with known concentrations of FeSO4 7H 2O. Results were expressed in µM (24).

### Nitric oxide assay

Griess method was used for determination of the serum levels of NO, which includes the conversion of nitrate to nitrite. Griess reagent facilitates the conversion of nitrite to a deep pink azo substance (25). Briefly, equal volumes of serum samples and Griess reagent were mixed and incubated at room temperature for 30-45 minutes. Next, the absorbance rate was determined at 540 and 630 nm using ELISA reader (STAT Fax 100, USA).

### Statistical analysis

All data were analyzed by SPSS software version 18.0 (Inc., Chicago, IL, USA) and presented as mean ± SE. Kolmogorov-Smirnov test was used to check the normality of the data. To compare the two groups, independent t test was used and Pearson correlation coefficient was used to determine the relationship between variables. The significance level was considered at P≥0.05.

## Results

In this study 80 women with a mean age of 31.36 ± 6.19 years were evaluated in two PCOS and control groups. The subjects were similar in age in both groups. Although the mean of BMI was higher in PCOS patients than in the control group, this difference was not statistically significant (Table 1).

**Fig 1:**
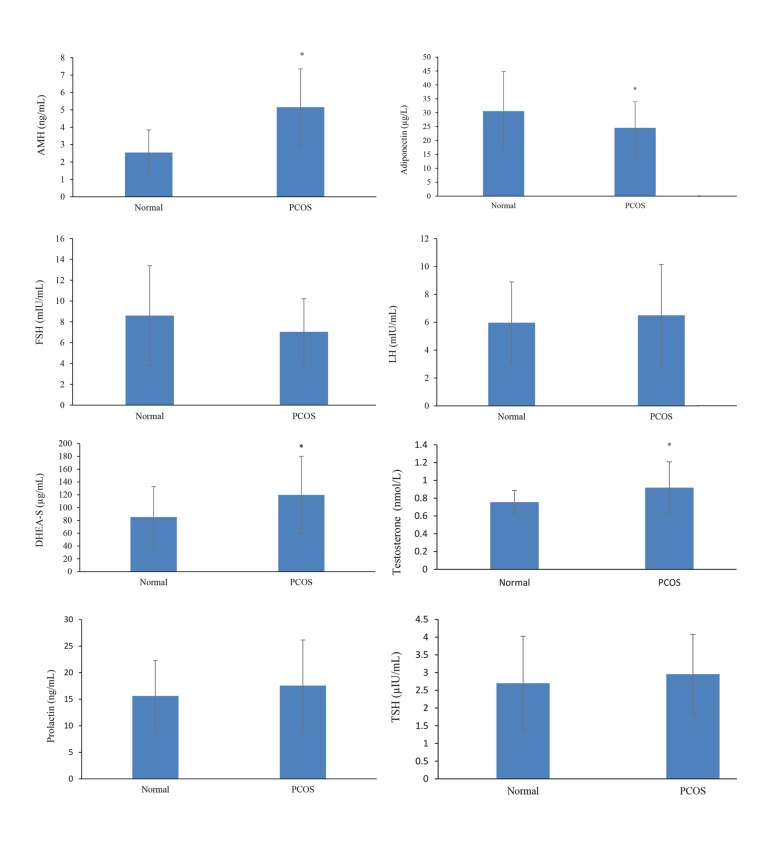
Comparison of the mean levels of AMH, Adiponectin and other hormones in control and PCOS groups. AMH; Anti-mullerian hormone, DHEA-S; Dehydroepiandrosterone sulfate, TSH; Thyroid stimulating hormone, FSH; Follicle-stimulating hormone, LH; Luteinizing hormone, and PCOS; Polycystic ovary syndrome. *; Significant difference between groups (P<0.05).

**Table 1:** Comparison of the mean levels of AMH, Adiponectin and other factors between control and PCOS groups


Variables	Control	PCOS	P value^*^

Age (Y)	32.02 ± 6.24	30.70 ± 6.14	0.341
BMI (Kg/m²)	25.33 ± 3.15	26.66 ± 4.24	0.117
AMH (ng/mL)	2.54 ± 2.44	5.16 ± 5.30	0.007
Adiponectin (µg/L)	30.57 ± 14.23	24.55 ± 9.41	0.029
DHEA-S (µg/mL)	85.25 ± 47.58	119.78±60.31	0.006
Testosterone (nmol/L)	0.76 ± 0.13	0.92 ± 0.29	0.002
Prolactin (ng/mL)	15.62 ± 6.66	17.57 ± 8.59	0.259
TSH (µIU/mL)	2.70 ± 1.32	2.96 ± 1.12	0.354
FSH (mIU/mL)	8.60 ± 4.79	7.04 ± 3.19	0.090
LH (mIU/mL)	5.97 ± 2.93	6.50 ± 3.65	0.476
FBG (mg/mL)	86.96 ± 9.52	85.63 ± 7.53	0.488
Insulin (µIU/mL)	5.68 ± 3.48	8.66 ± 3.98	0.001
IR-HOMA	1.25 ± 0.87	1.86 ± 0.90	0.003
TAC (µmol)	260.02 ± 212.71	231.26 ± 178.51	0.517
NO (µmol)	26.01 ± 12.41	31.11 ± 14.54	0.213


Data are presented as mean ± SD. AMH; Anti-mullerian hormone, PCOS; Polycystic ovary syndrome, BMI; Body mass index, DHEA-S; Dehydroepiandrosterone sulfate, TSH; Thyroid stimulating hormone, FSH; Follicle-stimulating hormone, LH; Luteinizing hormone, FBG; Fasting blood glucose, IR-HOMA; Insulin resistance- homeostatic model assessment, TAC; Total antioxidant capacity, NO; Nitric oxide, and *; Independent Sample t test.

### Biochemical analyzes

AMH level in PCOS group was significantly higher than in the normal group (5.16 ± 5.30 vs. 2.44 ± 2.49) (P=0.007). Also, there was a significant difference in the adiponectin level between the two groups (P=0.029), as it was lower in the PCOS group compared to the control group (24.55 ± 9.41 vs. 30.57 ± 14.23) (Table 1, Fig .1). There was no statistically significant difference in the mean of FSH and luteinizing hormone (LH) levels between the two groups, while the mean of both androgens in the PCOS group was significantly higher than in the control group (P=0.006 and P=0.002, respectively). Also, the mean of prolactin and TSH levels was higher in the PCOS group, but this difference was not significant (Table 1, Fig .1).

**Fig 2:**
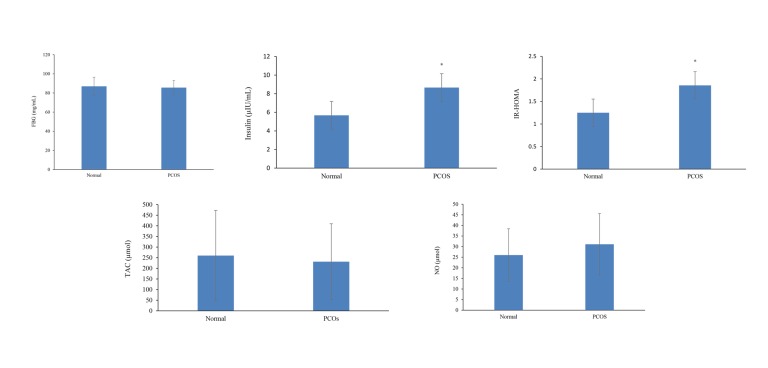
Comparison of insulin resistance index, TAC and NO in in control and PCOS groups. FBG; Fasting blood glucose, IR-HOMA; Insulin resistance-homeostatic model assessment, TAC; Total antioxidant capacity, NO; Nitric oxide, and PCOS; Polycystic ovary syndrome.*; Significant difference between groups (P<0.05.)

The mean of fasting blood glucose (FBG) was not significantly different between the two groups, but the mean of insulin in the PCOS group was significantly higher than in the control group (P=0.001). Also, the mean of insulin resistance-homeostatic model assessment (IR-HOMA) was significantly different between the two groups (P=0.003), It was higher in the PCOS group than control group (Table 1, Fig .2). Anti-oxidants and oxidative stress (OS) levels were evaluated in this study with two variables: TAC and serum NO. The mean of TAC was lower in the PCOS group and the mean of NO was higher than that of healthy subjects, but the difference was not statistically significant (Table 1, Fig .2).

### Correlation of variables in the PCOS patients

In the PCOS group, there was a significant negative correlation between age and AMH (P=0.002, r=-0.46), age and dehydroepiandrosterone sulfate (DHEA-S, P=0.045, r=-0.32), body mass index (BMI) and FSH (P=0.03, r=-0.34), and adiponectin and testosterone (P=0.02, r=-0.36). Also, There was a significant positive correlation between BMI and insulin (P=0.04, r=0.32) and IR (P=0.04, r=0.32), AMH and LH (P=0.10, r=0.4), DHEA-S and testosterone (P=0.003, r=0.45), DHEA-S and TAC (P=0.005, r=0.43), prolactin and nitric oxide (NO, P=0.04, r=0.42), and TSH and TAC (P=0.005, r=0.43). FBG (P=0.000, r=0.59) and insulin (P=0.000, r=0.99) also had a significant positive correlation with the IR index (IR-HOMA).

### Correlation between variables in the control subjects

In control subjects, there was a significant negative correlation between Age and AMH (P=0.000, r=- 0.76), Age and testosterone (P=0.01, r=-0.39), AMH and adiponectin (P=0.03, r=-0.35), and AMH and FSH (P=0.005, r=-0.43). There was a significant positive correlation between age and FSH (P=0.037, r=0.33). AMH and testosterone (P=0.01, r=0.39), prolactin and TAC (P=0.002, r=0.48), FBG and insulin (P=0.004, r=0.45), FBG and IR-HOMA (P=0.000, r=0.61), insulin and IR-HOMA (P=0.000, r=0.98), and IR-HOMA and NO (P=0.45, r =0.44). In the control subjects, increasing in BMI leads to decreasing in adiponectin (P=0.001, r=- 0.5) and DHEA-S (P=0.04, r=-0.34).

## Discussion

Several factors were studied in this study, but the most important results were the significant differences between AMH, adiponectin, androgens and IR between the two groups of PCOS patients and healthy controls. We observed significant correlations between these variables in the two groups independently. PCOS group showed biochemical features associated with PCOS, such as higher levels of androgens, insulin and IR. Also, there was a higher AMH and lower adiponectin level in PCOS patients. The most important correlation found in the PCOS group was a significant positive correlation between AMH and each of the factors LH, DHEA-S, TAC, prolactin, NO, BMI, insulin and IR. In addition, there was a significant negative correlation between AMH and DHEA-S, BMI and FSH, adiponectin and testosterone in the PCOS group. However, in the control group, there was a significant positive correlation between age and FSH, AMH and testosterone, prolactin and TAC, FBG and insulin, and IR and NO. On the other hand, there was a significant negative correlation between age and AMH, age and testosterone, BMI and adiponectin, BMI and DHEA-S, AMH and adiponectin, and AMH and FSH in this group.

The results of our study, similar to OlszaneckaGlinianowicz et al. (21), showed that PCOS as the most common endocrinopathology of women of reproductive age is accompanied with multiple metabolic changes, including increased androgen and insulin levels, and the emergence of IR. Many studies have shown that at least half of the people with PCOS are obese and that obesity plays a major role in the advent of IR in these individuals (15, 18). In our study, the mean of BMI of PCOS patients was higher than the control group but it was not significant. This finding is in contrast with the study of Woo et al. (26).

Adiponectin plays an important role in anti-inflammatory processes, insulin sensitivity and obesity. The results of some studies (16, 17), consistent with our study, show that adiponectin levels in PCOS patients are lower than in the healthy subjects, while in the study of Emadi et al. (27), there was not a significant difference in the level of adiponectin when comparing the two groups. Considering the prevalence of obesity in PCOS patients and the higher BMI in these subjects in our study, the lower mean of adiponectin and the increased mean of IR in this group was predictable.

In recent years, AMH has been used as a key factor for evaluating ovarian function and an indicator for determining the number of ovarian follicles and reverse. Due to the increase in the number of small follicles in the ovaries of PCOS patients, the increase of this hormone is not unexpected. In our study, after adjustment for age, AMH was significantly higher in the PCOS group, which was similar to the results of Woo et al. (26). Also, an increase in androgens and the number of follicles in PCOS group can lead to an increase the production of AMH, which may play a vital role in decreasing the sensitivity of growing follicles to FSH hormone. In the present study, similar to the findings of Mahdi et al. (28), the rate of androgens and AMH in the PCOS group is higher than in the control group, which may be due to an impairment in the production of AMH and androgens in these individuals. While in the control group with normal levels of androgens and AMH, there was a significant positive correlation between AMH and testosterone, which was similar to that of Woo et al. (26).

In some studies, the mean of FSH in patients with PCOS was higher than in the control group (26, 28), while in the present study, the mean of FSH was lower in the PCOS group. Nonetheless, similar to Hamza et al. (6) the difference that we observed was not significant. It can be suggested that increasing the number of small follicles and the AMH secreted from them, which lowers the sensitivity of the follicles to FSH, can affect the level of FSH and decrease its effect on PCOS patients. In a number of studies, levels of LH have increased dramatically in the follicular phase in PCOS patients. In our study, similar to Mahdi et al. (28), the mean of LH was higher in the PCOS group. Also, Hamza et al. (6) did not show any significant difference in the LH between the two groups.

There was a significant positive correlation between AMH and LH in the PCOS group and a significant negative correlation between AMH and FSH in the control group in the present study. In both groups the mean of AMH decreased with aging, which was similar to other previous studies (26-28). This decrease was due to a decrease in the number of follicles and ovarian reserves in women with an approach to menopause. It is also thought that with increasing age, the gonadotropins content in women should be increased (29). In our study, only in the control group age had a significant positive correlation with FSH. In the study by Swellam et al. (30), there was a significant correlation between age and decreasing of androgens in both groups, but in our study we did not find such correlation.

Although some studies have reported a negative correlation between AMH and BMI (31, 15), in our study, this correlation was not seen in either of the groups. Interestingly, the study by Nardo et al. (32) showed that AMH increased with increased activities of the subjects, and did not correlate with BMI. In our study we show that the BMI of PCOS individuals has a positive correlation with insulin level resistance, and a negative correlation with FSH. The results of various studies (17, 27) have shown that with increasing BMI, the levels of adiponectin in women decrease. However, in our study this was only observed in the control group.

In the present study, the correlation between AMH and adiponectin was negative in both groups, but it was significant only in the control group. In the study of Woo et al. (26), the correlation between AMH and adiponectin in the control group was direct and significant, which is the opposite of our findings; and in their PCOS group, there was no significant correlation between the two factors. In some of the previous studies (3, 6), in the PCOS group AMH has only a significant positive correlation with testosterone, which is different from our study results. The positive correlation between AMH and testosterone can be biologically normal for all women of reproductive age. These findings confirm that ovarian hyperandrogenesis has stopped the growth of follicles and in turn has increased AMH production. In our study, the absence of this association in the PCOS group may be due to a significant increase of the androgens and AMH in the PCOS patients, which can disturb the study of correlations.

Regarding prolactin, there is a hypothesis that polycystic ovaries affect the activity of dopamine in the hypothalamus and cause hyperprolactinemia in these patients (32). In our study, the level of prolactin in PCOS patients was higher than in healthy controls, and it was significantly correlated with an increase in NO levels, while in the control group there was a significant positive correlation between prolactin and TAC. In the present study the rate of IR in PCOS patients was higher than in the control group, similar to another previous study (17). Also, the correlation between IR and NO was found to be significant in the control group, which can be due to the effect of IR, which may also increase the level of oxidative stress in reducing ovulation (7).

The correlations between variables in the present study and the significant differences between the two groups can indicate the role of these factors in the pathogenesis of PCOS, which is a multifactorial disorder. More in depth research is needed for a better understanding of the molecular mechanism, cellular changes and gene expression that initiate PCOS pathogenesis.

## Conclusion

Adiponectin changes can lead to impaired ovarian function and ovarian hormones in the reproductive age and its deficiency in PCOS patients may be associated with IR and increased insulin levels. Insulin is one of the effective factors in increasing the number of antral follicles and ultimately increasing ovarian volume. In women suffering from PCOS hyperinsulinemia may increase AMH levels. So it can be concluded that the role of adiponectin in increasing insulin sensitivity plays a key role in controlling the synthesis of AMH in women of reproductive age.

## References

[1] March WA, Moore VM, Willson KJ, Phillips DI, Norman RJ, Davies MJ (2010). The prevalence of polycystic ovary syndrome in a community sample assessed under contrasting diagnostic criteria. Hum Reprod.

[2] Dunaif A (2008). Drug insight: insulin-sensitizing drugs in the treatment of polycystic ovary syndrome--a reappraisal. Nat Clin Pract Endocrinol Metab.

[3] Schuring AN, Schutie N, Sonntag B, Kiesel L (2008). Androgens and insulin-two key players in polycystic ovary syndrome.Recent concepts in the pathophsiology and genetics of polycystic ovary syndrome. Gynakol Geburtshilfliche Rundsch.

[4] Speroff L, Fritz MA (2005). Clinical gynecology endocrinology and infertility.

[5] Parco S, Novelli C, Vascotto F, Princi T (2011). Serum anti-Müllerian hormone as a predictive marker of polycystic ovarian syndrome. Int J Gen Med.

[6] Hamza SM (2016). Abd-alrahman SJ, Raheem SM.Correlation between levels of serum antioxidants and numerous hormones in primary infertility of women. Themed Section: Engineering and Technology.

[7] Diamanti-Kandarakis E, Piouka A, Livadas S, Piperi C, Katsikis I, Papavassiliou AG (2009). Anti-mullerian hormone is associated with advanced glycosylated end products in lean women with polycystic ovary syndrom. Eur J Endocrinol.

[8] Iwase A, Hirokawa W, Goto M, Takikawa S, Nagatomo Y, Nakahara T (2010). Serum anti Mullerian hormone level is a useful marker for evaluating the impact of laparoscopic cystectomy on ovarian reserve. Fertil Steril.

[9] Hsu MI (2013). Changes in the PCOS phenotype with age. Steroids.

[10] Iwase A, Sugita A, Hirokawa W, Goto M, Nakahara T, Bayasula (2013). Anti-mullerian hormone as a marker of ovarian reserve in patients with ovarian malignancies who have undergone fertilitypreserving surgery and chemotherapy. Gynecol Endocrinol.

[11] Peluso C, Fonseca FL, Rodart IF, Cavalcanti V, Gastaldo G, Christofolini DM (2014). AMH: An ovarian reserve biomarker in assisted reproduction. Clin Chim Acta.

[12] Kohzadi M, Choobsaz F, Khazaei M New findings on anti-mullerian hormone in polycystic ovarian syndrome patients. Gynecol Obstet.

[13] Alborzi S, Keramati P, Younesi M, Samsami A, Dadras N (2014). The impact of laparoscopic cystectomy on ovarian reserve in patients with unilateral and bilateral endometriomas. Fertil Steril.

[14] Farzadi L, Nouri M, Ghojazadeh M, Mohiti M, Aghadavod E (2012). Evaluation of ovarian reserve after laparoscopic surgery in patients with polycystic ovary syndrome. Bioimpacts.

[15] Goodarzi MO, Dumesic DA, Chazenbalk G, Aziz R (2011). Polycystic ovary syndrome: etiology, pathogenesis and diagnosis. Nat Rev Endocrinol.

[16] Viengchareun S, Zennaro MC, Pascual-Le Tallec L, Lombes M (2002). Brown adipocytes are novel sites of expression and regulation of adiponectin and resistin. FEBS Lett.

[17] Corbould A, Kim YB, Youngren JF, Pender C, Kahn BB, Lee A (2005). Insulin resistance in the skeletal muscle of women with PCOS involves intrinsic and acquired defects in insulin signalling. Am J Physiol Endocrinol Metab.

[18] Bohlouli S, Rabzia A, Sadeghi E, Chobsaz F, Khazaei M (2016). In vitro anti-proliferative effect of adiponectin on human endometriotic stromal cells through adipoR1 and adipoR2 gene receptor expression. Iran Biomed J.

[19] Bohlouli S, Khazaei M, Rabzia A, Khazaei MR, Sadeghi E (2015). Adiponectin effect on nitric oxide secretion by normal and endometriotic human endometrial stromal cells: in vitro study. Int J Morphol.

[20] Silvestris E, de Pergola G, Rosania R, Loverro G (2018). Obesity as disruptor of the female fertility. Reprod Biol Endocrinol.

[21] Olszanecka-Glinianowicz M, Madej P, Owczarek A, Chudek J, Skałba P (2015). Circulating anti-Müllerian hormone levels in relation to nutritional status and selected adipokines levels in polycystic ovary syndrome. Clin Endocrinol (Oxf).

[22] Montazerifar F, Karajibani M, Ghasemi M, Khorram Rouz F, Alsadat Hosseini F, Bagheri N (2016). Evaluation of association between serum leptin and adiponetin levels with obesity markers, lipid profile and hormonal parameters in women with polycystic ovary syndrome. Middle-East J Sci Res.

[23] Rotterdam ESHRE/ASRM-Sponsored PCOS consensus workshop group (2004). Revised 2003 consensus on diagnostic criteria and longterm health risks related to polycystic ovary syndrome (PCOS). Hum Reprod.

[24] Ghanbari E, Nejati V, Khazaei M (2016). Antioxidant and protective effects of royal jelly on histopathological changes in testis of diabetic rats. Int J Reprod Biomed.

[25] Khazaei M, Pazhohi M, Khazaei S (2018). Evaluation of hydro-alcoholic extract of Trifolium pratens L.for its anti-cancer potential on U87MG cell line. Cell J.

[26] Woo HY, Kim KH, Rhee EJ, Park H, Lee MK (2012). Differences of the association of anti-Müllerian hormone with clinical or biochemical characteristics between women with and without polycystic ovarian syndrome. Endocr J.

[27] Emadi M, Ramezani Tehrani F, Yaghmaei P, Sheikholeslami S, Hedayati M (2012). Serum adiponectin levels and its association with insulin resistance and obesity in women with poly cystic ovarian syndrome. RJMS.

[28] Mahdi WKM, Mohammed MS, Sanad AS (2016). Association of polycystic ovary syndrome and adiponectin gene polymorphisms. Arch Clin Microbiol.

[29] Rodrigues MA, Verdile G, Foster JK, Hogervorst E, Joesbury K, Dhaliwal S (2008). Gonadotropins and cognition in older women. J Alzheimers Dis.

[30] Swellam M, Khaial A, Mosa T, El-Baz H, Said M (2013). Anti-mullerian and androgens hormones in women with polycystic ovary syndrome undergoing IVF/ICSI. Iran J Reprod Med.

[31] Buyuk E, Seiferet DB, Illions E, Grazi RV, Liemen H (2011). Elevated body mass index is associated with lower serum anti-mullerian hormone levels in infertile women with diminished ovarian reserve but not with normal ovarian reserve. Fertil Steril.

[32] Nardo LG, Yates AP, Roberts SA, Pemberton P, Laing I (2009). The relationships between AMH, androgens, insulin resistance and basal ovarian follicular status in non-obese subfertile women with and without polycystic ovary syndrome. Hum Reprod.

